# The Hospital Library and Charities Bureau

**Published:** 1904-04-02

**Authors:** 


### THE HOSPITAL LIBRARY AND CHARITIES BUREAU.

Inquiries of the librarian are answered in this column free of charge. Inquiries to be answered promptly by post must be accompanied by a fee of 2s. 6d.

*Anon* writes:—Are ladies admissible upon the Boards of Management of hospitals?

Yes. The practice is becoming very general, especially in the case of hospitals for women and children, small hospitals, and cottage hospitals. Many hospitals have a separate ladies' committee.

